# The complex interplay between sectoral energy consumption and economic growth: Policy implications for Iran and beyond

**DOI:** 10.1016/j.heliyon.2024.e31988

**Published:** 2024-05-28

**Authors:** Hesam Ghadaksaz, Yadollah Saboohi

**Affiliations:** a Department of Mechanical Engineering, Aalto University, Espoo, Finland; b Department of Energy Engineering, Sharif University of Technology, Tehran, Iran

**Keywords:** Sectoral energy consumption, Economic growth, Cointegration and causality analysis, Iran

## Abstract

Iran's abundant energy reserves starkly contrast with recent power and gas shortages, particularly impacting the industrial sector. Furthermore, long-term trends reveal a concerning pattern where total primary energy consumption has outpaced economic growth, doubling in recent decades. These challenges emphasize the need for a thorough evaluation of the intricate interplay between sectoral energy consumption and economic output in Iran, bearing profound policy implications. The current study employs ARDL and VECM approaches to analyze empirical long- and short-term dynamics. Regarding Iran, the results unveil causal relationships from industrial energy consumption to GDP and from GDP to energy consumption in buildings. Notably the significant positive value of elasticity of GDP with respect to industrial energy use highlights the need for nuanced energy management measures. Variations across sectors underscore the justification for recognizing industrial energy consumption as productive energy use. The results gain additional support from a panel data analysis spanning fourteen diverse countries, bearing significance for IAMs applied in climate change research. While IAMs traditionally employ total energy consumption or the sectoral energy uses collectively, as production factors, the research highlights the need to reevaluate model frameworks for potential different outcomes from established practices.

## 1 Introduction

Iran, despite its abundant natural gas and other energy resources, grapples persistently with excessive energy consumption due to glaring inefficiencies. The winter of 2023 stands as a stark reminder, as authorities in several regions were compelled to close schools and government offices for extended periods to conserve natural gas. Similarly, prioritizing residential household electricity over industrial needs during scorching summer weeks disrupted the operational continuity of manufacturing firms.

**Fig. 1:**
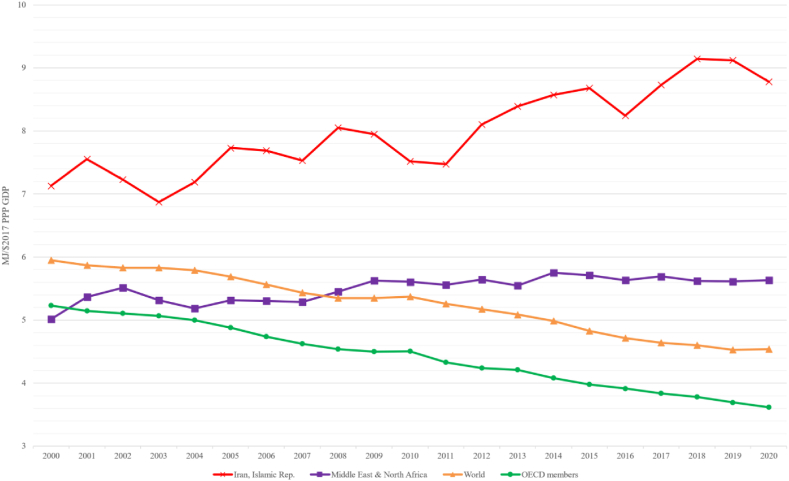
Energy intensity levels of primary energy across Iran, MENA, World, and OECD members (2000–2020) [1].

These immediate energy shortage concerns are compounded by long-term systemic challenges in the realm of energy intensity and climate change. Iran finds itself among the top 10 nations worldwide in terms of greenhouse gas (GHG) emissions [1], raising significant environmental and climate change concerns. This is particularly concerning given the global trend of diminishing energy intensity and the emergence of decoupling between economic growth and energy consumption, in many cases, in both developed and, to varying extents, developing countries over recent years. However, Iran exhibits a contrary trajectory (Fig. 1), where energy intensity has been on the rise. An in-depth examination of official statistics spanning various timeframes, including the past 10, 30, and 50 years, unveils a striking pattern: the total primary energy consumption in Iran has grown at a rate twice as swift as the economic output [2]. The confluence of immediate energy supply challenges and systemic complexities within Iran's energy landscape serves as the impetus driving the present study, which aims to comprehensively analyze the nuanced relationship between energy consumption and economic performance [3]. The study endeavors to dissect the intricate relationship between energy consumption and economic output within Iran, utilizing the autoregressive distributed lag (ARDL) and vector error correction model (VECM) approaches within a multivariate framework, thereby offering an empirical lens to explore these pressing concerns.

In addition to offering policy recommendations on sustaining the development of the energy system in Iran, the research outcomes hold the potential to reevaluate the core formulations of the dynamics between energy use and economic output within the integrated assessment modeling studies. Nowadays, integrated assessment models are extensively used as crucial tools for gauging the technological and economic viability of climate-related interventions. Prominent among these models are MARKAL/TIMES-MACRO [4] and MESSAGE-MACRO [5], which rest on the premise that factors including capital stock, labor, and energy collectively dictate overall economic output, encapsulated within a nested production function. However, it's imperative to recognize that these models traditionally treat energy consumption in non-productive sectors, such as residential buildings, as a contributing factor to economic value-added. This perspective on the role of energy use in economic production would differ from the nuanced insights gained through cointegration analysis, which delves into the intricate association between sectoral energy consumption and economic growth. The current novel study, unlike many prior studies that primarily investigate total energy consumption or energy consumption across various carriers, prompts a reevaluation of production functions and the iterative information exchange inherent in integrated modeling practices. To reinforce the robustness of the recommendation to regard specific sectoral energy use as production factor, an extensive examination is also conducted across fourteen different economies, chosen for their geographical diversity.

Also, adopting an alternative analytical approach, the conventional concept of energy is substituted with exergy, offering a unique perspective for evaluating its capacity to provide deeper insights into the underlying dynamics. Exergy, which captures the capability of a unit of energy to perform useful work, is used to determine whether it offers a more effective explanatory framework compared to traditional energy metrics.

By exploring these diverse analytical avenues, the current research extends beyond its specific focus on Iran and contributes valuable insights to the broader discourse surrounding studies at the intersection of energy, economy, and climate considerations. Since the pivotal study by J. Kraft and A. Kraft [6], numerous cointegration and causality analyses have been done to investigate how critical is the role of energy consumption in economic growth. The papers by I. Ozturk [7], J. E. Payne [8], N. Apergis and J.E. Payne [9], R. Smyth and P.K. Narayan [10], M. Azam et al. [11], B. N. Iyke [12], and Md. S. Rahman et al. [13] summarize the vast body of literature regarding the proposed countries, intended (proxy) variables, applied econometric methods, and even the results.

Table 1 offers a succinct overview of prior studies in the context of Iran. A comprehensive review of the literature underscores the absence of consistent and comparable results across these various investigations. The divergent findings documented in these studies can be attributed to a range of influential factors, including disparities in the economic development stage of the country, variations in the datasets employed, differences in model specifications, and the use of diverse econometric methodologies [7,8,10,14].

**Table 1:** Summary of studies regarding the energy and economic growth relationship in Iran.

Authors	Method	Timespan	Results
M. Zamani [19]	vector error correction	1967 to 2003	long-run unidirectional relationship from GDP to total energy consumption
J. Squalli [20]	Toda Yamamoto and ARDL	1980 to 2003	bi-directional positive causality between per capita electricity consumption and real per capita GDP
P.K. Narayan and S. Popp [21]	ARDL	1980 to 2006	neutrality hypothesis on the long-run relationship between energy consumption and real GDP
M. Mehrara [22]	panel cointegration analysis	1971 to 2002	energy conservation through reforming energy price policies has no damaging repercussions on economic growth
N. Apergis and C.F. Tang [23]	Toda-Yamamoto-Dolado-Lütkepohl Granger causality	1975 to 2007	energy consumption Granger causes economic growth
C.C. Lee and C.P. Chang [24]	panel-based error correction	1971 to 2002	a significant positive impact of energy consumption on GDP
S. Nasreen and S. Anwar [25]	panel cointegration and causality	1980 to 2011	income has a positive and significant impact on energy consumption
N. Ahmad and L. Du [26]	ARDL	1971 to 2011	energy generation has a positive and significant effect on economic growth in the short- and long-run
M.R. Lotfalipour et al. [27]	Toda-Yamamoto	1967 to 2007	unidirectional long-run Granger causality from real GDP to the total consumption of petroleum products and other fossil fuels
M.S. Gorus and M. Aydin [28]	Granger causality analysis	1975 to 2014	conservation hypothesis holds in the short-run, while the growth hypothesis is valid in the long-run
S. Erdogan et al. [29]	panel data analysis within a bivariate framework	1990 to 2014	nither energy consumption causes economic growth, nor economic growth causes energy consumption
P.K. Narayan and R. Smyth [30]	panel causality analysis	1974 to 2002	statistically significant feedback effects between electricity consumption, exports, and GDP

This study distinguishes itself from prior research on Iran by incorporating a comprehensive set of variables, including GDP, capital, labor, and trade openness, while also considering segmented energy use, in line with established theories of economic growth. The conventional approach of focusing on individual energy carriers separately or aggregated in empirical investigations may lead to distorted results, given that energy resources and carriers tend to be subject to substitution over time. Therefore, the present study deviates from the predominant approach in previous research [[15], [16], [17], [18]] by not concentrating on individual or aggregated energy resources.

The subsequent sections of this paper are structured as follows: section 2 outlines the employed data and methodology, section 3 presents the empirical findings and initiates a comprehensive discussion, which also encompasses an examination of the robustness of the results. Lastly, section 4 concludes the study, summarizing the principal findings and delineating their policy implications.

## 2 Model specification and data sources

In terms of model specification, the literature can be categorized into two distinct strands. The first strand, encompassing earlier studies, primarily employed bivariate models to examine the relationship between energy and economic growth, or their proxy variables [6,14,16,[19], [20], [21], [22],[31], [32], [33], [34], [35], [36], [37], [38], [39], [40], [41], [42], [43], [44], [45], [46], [47], [48], [49], [50], [51], [52], [53]]. However, it is recognized that omitting relevant variables in such models can lead to biased conclusions regarding causal inferences [54]. The second strand of the literature delves into the causal relationship between energy and economic growth within a multivariate framework, incorporating one or more additional variables. This expanded approach is intended to provide a more comprehensive understanding of the intricate dynamics at play. Some studies, for instance, have introduced variables such as capital formation or labor [9,15,17,18,23,24,[55], [56], [57], [58], [59], [60], [61], [62], [63], [64], [65], [66], [67], [68], [69], [70], [71], [72], [73], [74]] into the model. Others have expanded the model by considering urbanization [[75], [76], [77], [78], [79]], international trade [25,80], and various other related factors, including energy prices, foreign direct investment, weather-based indices, political circumstances, consumer price indices, inflation rates, resource rents, and political structures [11,12,[81], [82], [83], [84], [85], [86], [87], [88], [89]]. These multifaceted approaches serve to enrich the empirical examination of the energy-economic growth relationship, shedding light on the potential interplay of a myriad of influencing factors.

The inclusion of labor and capital in the relationship between energy and economic growth is underpinned by a sound theoretical foundation. Mainstream growth models typically do not recognize energy as a fundamental factor of production, while models rooted in ecological economics emphasize energy as the primary factor, often neglecting the role of other conventional factors. However, there is a perspective that seeks to bridge these two approaches [90]. Considering the objectives of the present study, a model specification rooted in this integrated approach is applied.

Table 2 outlines the model specifications typically used in similar studies. It is noteworthy that, in the past, many studies assumed exogenous technological improvements in their models. More recent research has aimed to proxy technological progress through considerations such as time trends, exports/imports, or financial development [83,91]. Trade openness, in particular, has garnered attention as it fosters competition in both domestic and foreign markets, promotes efficient resource utilization, and facilitates the dissemination of knowledge and technology [92]. The diffusion of technology through international trade engenders spill-over benefits, which can significantly benefit developing and less developed nations.

**Table 2:** Summary of primary studies on energy and economy nexus based on the neoclassical growth theory.

Authors	Model specification^a^	Method	Conclusion
N. Apergis and J.E. Payne [9]	Y = f(ELC, K, L)	vector error correction	varied relationship across 88 countries with different income levels
N. Apergis and J.E. Payne [62]	Y = f(t, RE, K, L)	vector error correction	bidirectional causality between renewable energy consumption and economic growth in both the short- and long-run in six Central American countries
C. Pirlogea and C. Cicea [63]	Y = f(E, K, L)	ARDL	long-run relationships between GDP per capita and consumption of renewables and total petroleum products in Spain, Romania, and the European Union
M. Shahiduzzaman and K. Alam [64]	Y = f(t, E, K, L)	Toda and Yamamoto	bidirectional relationship between GDP and energy use in Australia
M. Shahbaz and H.H. Lean [65]	Y = f(E, K, L)	ARDL	bidirectional relationship between electricity consumption and economic growth in Pakistan
H. Heidari et al. [66]	Y = f(GAS, K, L)	ARDL	bidirectional positive relationship in both the short- and long-run in Iran
A. Ohler and I. Fetters [67]	Y = f(K, L, RELG, NRELG)	vector error correction	bidirectional relationship between aggregate renewable generation and real GDP across twenty OECD countries
M. Shahbaz et al. [68]	Y = f(RE, K, L)	ARDL and rolling window	long-run bidirectional relationship between economic growth and renewable energy consumption in Pakistan
Z. Fang and Y. Chang [70]	Y = f(E, H, K, L)	Cup-FM and FMOLS	long-run relationship and Granger causality from economic growth to energy use in Asia Pacific Countries
E. Kocak and A. Sarkgunesi [71]	y = f(RE, K, L)	DOLS and FMOLS	renewable energy consumption has a significant impact on economic growth in the Black Sea and Balkan region countries
W. Saad and A. Taleb [72]	Y = f(RE, K, L)	vector error correction	presence of unidirectional causality from economic growth to renewable energy consumption in the short-run and a bidirectional causal relationship in the long-run in 12 European Union countries
J.H. Yuan et al. [18]	Y = f(K, L, E)	Johansen	unlike Granger-causality outcomes across different energy carriers in China
N. Bowden and J.E. Payne [56]	Y = f(IE, RE, CE, TE, E, K, L)	Toda–Yamamoto	different relationships between real GDP and energy consumption across sectors in the US.
M. Bartleet and R. Gounder [58]	Y = f(E, EP); Y = f(E, L); Y = f(E, L, K)	ARDL	Granger causality from real GDP to energy consumption in New Zealand
B.S. Warr and R.U. Ayres [59]	Y = f(K, L, EX); Y = f(K, L, UW)	vector error correction	output growth does not drive energy consumption in US.
Y. Wang et al. [60]	Y = f(K, L, E)	ARDL	existence of short-run and long-run causality running from energy consumption to economic growth in China
M. Shahbaz et al. [83]	Y = f(K, L, E, TR, FD)	ARDL	a long-run relationship and the unidirectional causality running from energy use to economic growth in China
P. Sadorsky [91]	Y = f(K, L, E, TR)	vector error correction	a causal relationship between energy and exports/imports in the long run in seven South American countries

a Y, y, K, L, H, E, IE, RE, CE, TE, EX, UW, ELC, RE, RELG, NRELG, GAS, EP, TR, FD, and t refer to (real) GDP, per capita GDP, gross (fixed) capital formation/capital stock, total labor force, human capital (based on years of schooling), energy consumption, industrial energy consumption, residential energy consumption, commercial energy consumption, transportation energy consumption, total exergy consumption, total useful work consumption, electricity consumption, renewable energy consumption, renewable electricity generation, non-renewable electricity generation, natural gas consumption, energy price, trade, financial development, and period, respectively.

Consequently, the current research introduces trade openness into the model specification as a proxy for technological progress, thereby endogenizing technological advancement. Trade openness is measured as the combined value of exports and imports of goods and services, expressed as a proportion of the Gross Domestic Product. It is important to acknowledge that trade openness also reflects revenue from oil exports. Additionally, in the case of Iran, trade openness may concurrently mirror the effects of economic sanctions, as the imposition of such sanctions can significantly curtail trade levels.

Within the conceptual framework of the energy system, which encompasses the entire energy chain from energy resources to final energy carriers to energy services, final energy carriers are utilized through various technologies to provide energy services, also referred to as useful energy or useful work. Hence, the notion of considering useful work as a driving variable, applied in some previous studies [93], does not align well with theoretical foundations. Consequently, it is more theoretically appropriate to view final energy, in conjunction with labor, capital, and productivity, as the driving forces behind economic growth. Specifically, in the context of this research, sectoral final energy consumption is selected as the appropriate energy variable. By distinguishing between energy use in buildings and industrial energy use and employing the proposed model specification, the study aims to investigate the hypothesis that productive energy consumption, which can be substituted by capital, labor, and trade openness, contributes to value-added economic growth. To mitigate potential biases in causality analysis, earlier work by Pablo-Romero and Sanchez-Braza [94] introduced the concept of productive energy. Productive energy refers to the total final energy consumption in productive processes, while excluding other energy uses. This distinction is valuable in disentangling the economic implications of energy consumption in different contexts and sectors, offering a more nuanced perspective on its role in driving economic growth.

**Fig. 2:**
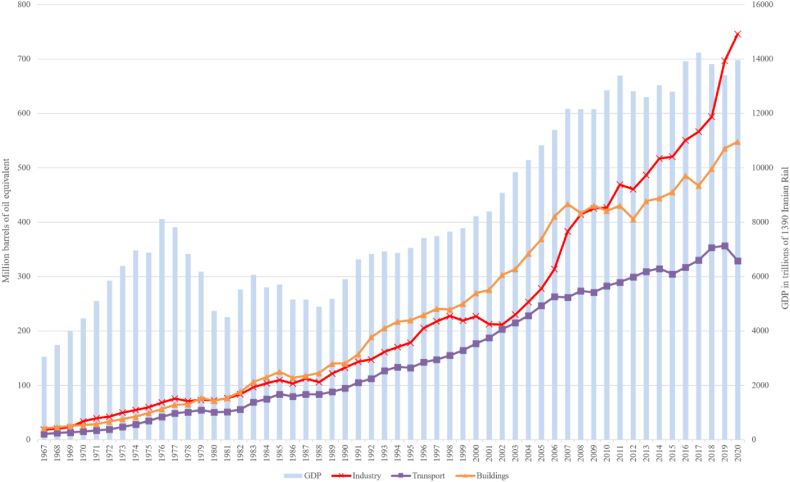
Trends in GDP and sectoral final energy consumption in Iran (1967–2020) [2].

Data used in the present research are annual time series from 1967 to 2018, taken from the World Bank Data [1], Central Bank of Iran [95], Iran Energy Statistics Yearbook 2018 [96], and IEA [97]. The proposed variables are GDP (Y) and capital stock (K), the labor force (L), aggregated and sectoral final energy consumption (E), and trade openness (TO). The availability of energy-related data constrained the starting point selection in 1967. Fig. 2 presents GDP and final energy consumption trends in Iran over the past fifty years. As reported in the Iran Energy Statistics Yearbook 2018, the share of buildings, industrial, transportation, and agriculture sectors in final energy consumption is 34.5 %, 36.8 %, 24.4 %, and 4.0 %, respectively [2].

## 3 Methodology

The empirical examination of the relationship between energy use and economic production underscores intricate dynamics and the necessity for robust analytical methods to accurately disentangle these relationships. Traditional linear regression or correlation methods are inadequate for establishing causal relations among variables trending over time, as shared directionality can lead to spurious correlations. Instead, causality tests and cointegration analysis are essential tools in energy economics research, allowing for rigorous examination of causal relationships and long-term equilibrium dynamics [98].

The empirical model used to explore the long-term relationship between energy consumption and economic output is presented as follows:

1 $$f\left(\ln\;Y_{t},\ln\;E_{t},\ln\;K_{t},\ln\;L_{t},\ln\;{TO}_{t}\right)=0$$

Here, lnY_t_, lnK_t_, lnL_t_, and lnTO_t_ are the natural logarithm of GDP, capital stock, labor force, and trade openness, respectively. As well, lnE_t_ represents aggregated or sectoral energy consumption. The use of natural logarithms for these variables is a statistical technique that helps mitigate the impact of outliers and aligns with the assumptions of inferential statistics. An alternative specification has also been developed in which energy consumption (lnE_t_) is substituted with exergy consumption (lnEx_t_). In this context, exergy is employed to account for the quality of energy and its capacity to perform useful work, offering a distinct perspective on the relationship between energy and economic growth by focusing on the efficiency and quality of energy in driving economic processes. Well-defined coefficients are utilized to convert the thermal energy content of different energy carriers into their exergy content [59]. For instance, electricity is commonly considered as pure useful work, with an energy to exergy conversion rate of one.

In this study, ARDL bounds testing [99,100] and VECM Granger causality approaches are used to investigate cointegration and causality, respectively. The analysis unfolds in several steps. Initially, a unit root test is conducted to ascertain whether the variables under consideration exhibit a unit root, indicating non-stationarity. Following this, the ARDL approach is employed to investigate the presence of a long-run equilibrium relationship among the variables of interest. This step aims to assess whether these variables have a stable, consistent relationship over the long term. Subsequently, multivariate Granger-type causality tests are constructed within the framework of a VECM. This step allows for the determination of the direction of causality among the variables, shedding light on how they influence each other. By employing these methodologies, the study seeks to establish the presence of cointegration and the causal relationships among the variables in question.

### 3.1 Unit root test

Before conducting cointegration tests, it is imperative to assess the stationarity of the time series data. The ARDL modeling approach does not impose a strict requirement for all variables to be integrated of order 0, I(0), or 1,I(1), but it's important to note that the critical F-statistics used for cointegration analysis are not valid if any variable in the model is integrated of order two, I(2), or higher.

To ensure robust results, this study employs two different unit root tests: the augmented Dickey-Fuller (ADF) and Phillips-Perron (PP) tests. Both tests aim to evaluate the null hypothesis, suggesting the presence of non-stationarity, against the alternative hypothesis, which indicates the absence of a unit root (stationarity). By conducting both ADF and PP tests, the study seeks to enhance the reliability and robustness of the stationarity assessments.

### 3.2 Cointegration test

The ARDL bounds testing approach is proposed to examine the long-run relationship among the variables. The ARDL model has a general form where the dependent variable in difference form can depend on itself (in lagged level or differences) and other dependent variables, which may be in lagged levels, contemporaneous differences, or lagged differences. In the context of this study, the following models are to be estimated within the ARDL framework, allowing for an extensive examination of the relationships among the variables:

2 $$\Delta \ln\;Y_{t}=\alpha_{0}+\sum_{i=1}^{\text{lYY}}\alpha_{\text{Yi}}\Delta \ln\;Y_{t‐i}+\sum_{i=0}^{\text{lYE}}\alpha_{\text{Ei}}\Delta \ln\;E_{t‐i}+\sum_{i=0}^{\text{lYK}}\alpha_{\text{Ki}}\Delta \ln\;K_{t‐i}+\sum_{i=0}^{\text{lYL}}\alpha_{\text{Li}}\Delta \ln\;L_{t‐i}+\sum_{i=0}^{\text{lYT}}\alpha_{\text{Ti}}\Delta \ln\;{\text{TO}}_{t‐i}+\alpha_{Y}\;\ln\;Y_{t‐1}+\alpha_{E}\;\ln\;E_{t‐1}+\alpha_{K}\;\ln\;K_{t‐1}+\alpha_{L}\;\ln\;L_{t‐1}+\alpha_{T}\;\ln\;{\text{TO}}_{t‐1}+\varepsilon_{\text{Yt}}$$

3 $$\Delta \ln\;E_{t}=\beta_{0}+\sum_{i=1}^{lEE}\beta_{Yi}\Delta \ln\;E_{t‐i}+\sum_{i=0}^{lEY}\beta_{Ei}\Delta \ln\;Y_{t‐i}+\sum_{i=0}^{lEK}\beta_{Ki}\Delta \ln\;K_{t‐i}+\sum_{i=0}^{lEL}\beta_{Li}\Delta \ln\;L_{t‐i}+\sum_{i=0}^{lET}\beta_{Ti}\Delta \ln\;{TO}_{t‐i}+\beta_{Y}\;\ln\;Y_{t‐1}+\beta_{E}\;\ln\;E_{t‐1}+\beta_{K}\;\ln\;K_{t‐1}+\beta_{L}\;\ln\;L_{t‐1}+\beta_{T}\;\ln\;{TO}_{t‐1}+{\;\varepsilon }_{Et}$$

The selection of the optimal lag orders is guided by minimizing the values of the Akaike Information Criterion (AIC). Due to the limited number of observations, a maximum of four lags was employed in this process. Moreover, the study utilizes several diagnostic tests to assess the model's reliability and robustness. These tests include the Shapiro-Wilk test for normality, the Breusch-Godfrey (BG) test for serial correlation, the Breusch-Pagan (BP) test for heteroscedasticity, and Ramsey's RESET test for potential functional form misspecification. These diagnostic tests help ensure the model's reliability and robustness by assessing its adherence to key statistical assumptions, such as normality, absence of serial correlation, homoscedasticity, and correct functional form specification.

The null hypothesis of no long-run relationship between the variables in Equation (2) is H_0_: α_j_ = 0, j = Y, E, K, L, T against the alternative hypothesis of cointegration H_1_: α_j_ ≠ 0, j = Y, E, K, L, T. Similar hypotheses can be derived for Equation (3). MH Pesaran et al. [101] generated two sets of critical values as the upper and lower bound critical values. If the calculated F-statistics lie above the band's upper level, the null hypothesis is rejected, indicating cointegration. If the F-statistics is below the lower critical value, the null hypothesis of no-cointegration cannot be rejected. Finally, the decision regarding cointegration is inconclusive if the calculated F-statistic falls between the two critical values.

### 3.3 Causality test

The third step in the analysis involves constructing Granger-type causality models augmented with a lagged error correction term (ECT), given that the series are cointegrated. In cases where no cointegration is detected, an ARDL short-run model is developed to investigate short-run causality. The Granger causality approach is applied to examine the direction of causality among the variables. The equations used for the causality analysis are presented in Equations 4, 5). In these equations, the significance of the coefficients of the differenced terms serves to establish the short-term dynamics from any independent variable to the dependent variable. Furthermore, the existence of a long-term relationship is contingent on the statistical significance of the lagged error correction term, ECT_t-1_, with a negative sign, underscoring the role of the error correction mechanism in restoring equilibrium over time.

4 $$\Delta \ln\;Y_{t}=\gamma_{0}+\sum_{i=1}^{lyy}\gamma_{Yi}\Delta \ln\;Y_{t‐i}+\sum_{i=0}^{lye}\gamma_{Ei}\Delta \ln\;E_{t‐i}+\sum_{i=0}^{lyk}\gamma_{Ki}\Delta \ln\;K_{t‐i}+\sum_{i=0}^{lyl}\gamma_{Li}\Delta \ln\;L_{t‐i}+\sum_{i=0}^{lyt}\gamma_{Ti}\Delta \ln\;{TO}_{t‐i}+\gamma_{ect}{ECT}_{t‐1}+\epsilon_{Yt}$$

5 $$\Delta \ln\;E_{t}=\delta_{0}+\sum_{i=1}^{lee}\delta_{Yi}\Delta \ln\;E_{t‐i}+\sum_{i=0}^{ley}\delta_{Ei}\Delta \ln\;Y_{t‐i}+\sum_{i=0}^{lek}\delta_{Ki}\Delta \ln\;K_{t‐i}+\sum_{i=0}^{lel}\delta_{Li}\Delta \ln\;L_{t‐i}+\sum_{i=0}^{let}\delta_{Ti}\Delta \ln\;{TO}_{t‐i}+\delta_{ect}{ECT}_{t‐1}+\epsilon_{Et}$$

The incorporation of ECT implies that the fluctuations in the endogenous variables are influenced by the extent of deviation from the long-term equilibrium. Therefore, the coefficients associated with these error correction terms signify the degree of disparity or divergence of the dependent variables from their long-run equilibrium values. In essence, ECT serve as a mechanism to correct or adjust for any deviations from the long-term balance in the model, facilitating an understanding of how the system returns to equilibrium following temporary imbalances.

## 4 Results and discussion

This section presents a comprehensive presentation of the study's findings, commencing with the outcomes of the unit root tests. Subsequently, it delves into the ARDL bounds testing and Granger-causality analyses. In the initial phase, the Augmented Dickey-Fuller (ADF) and Phillips-Perron (PP) tests for the presence of a unit root were executed on both the level and first-difference terms of the time series data, employing a significance level of five percent. The results of the stationarity tests are displayed in Table 3, where lnEI, lnEB, and lnET correspond to the natural logarithms of final energy consumption in the industrial, buildings, and transportation sectors, respectively. According to the findings from the ADF and PP tests, all variables exhibit stationarity after a single differencing operation. With the stationarity of the variables established, the subsequent step involves the application of the ARDL bounds testing method to scrutinize the existence of a long-term relationship among the targeted variables.

**Table 3:** The number of differences required for time series to be stationary.

statistics	ADF	PP
lnY	1	1
lnK	0	0
lnTO	1	1
lnL	1	1
lnE	0	0
lnEI	1	1
lnEB	0	0
lnET	0	0
lnEx	0	0
lnExI	1	1
lnExB	0	0
lnExT	0	0

### 4.1 Cointegration evidence

The analysis results for models with computed F-statistics that exceed the upper bound critical value at a 5 % significance level, demonstrating valid diagnostic statistics, are presented in Table 4. Selecting the appropriate lag length before applying the ARDL bounds testing approach is a crucial preliminary step, ensuring that the analysis is based on a robust and reliable framework.

**Table 4:** The results of the ARDL cointegration test with asymptotic critical value bounds of case III.

Energy-Economy relationship^a^	lnE ∼ lnY	lnY ∼ lnEI	lnY ∼ lnExI	lnEB ∼ lnY	lnExB ∼ lnY	lnY ∼ lnET	lnET ∼ lnY	lnExT ∼ lnY
Optimal lag length	(3,3)	(4,3)	(4,3)	(4,4)	(4,4)	(4,3)	(4,4)	(2,2)
F-statistic	6.198	7.667	7.765	5.757	5.477	4.655	4.995	5.547
Critical values for F-statistic	2.578 and 3.71 as 10 % lower and upper critical values, respectively 3.068 and 4.334 as 5 % lower and upper critical values, respectively 4.244 and 5.726 as 1 % lower and upper critical values, respectively							
Adj. R-Squared	0.789	0.792	0.793	0.780	0.764	0.806	0.864	0.797
Ramsey's RESET^b^	0.204	0.173	0.206	0.727	0.625	0.637	0.416	0.140
BG statistic^b^	0.3013	0.519	0.527	0.541	0.724	0.836	0.610	0.746
BP statistic^b^	0.427	0.343	0.308	0.214	0.198	0.757	0.259	0.791
Shapiro-Wilk statistic^b^	0.777	0.972	0.967	0.547	0.756	0.486	0.682	0.119

a All the specifications consider capital, labor, and trade openness as independent variables besides the mentioned one.

b in *p*-values.

Cointegration is observed when GDP is considered as the dependent variable. It occurs when either final energy consumption in the industry sector (EI) or transportation sector (ET), along with capital stock, labor force, and trade openness, are the driving variables. Additionally, cointegration is found when total final energy consumption and final energy consumption in the buildings and transportation sectors are considered as the dependent variables, with GDP, capital, labor, and trade openness as the driving variables.

It is important to note that the long-term relationships of lnY ∼ lnET and lnET ∼ lnY are significant at the 5 % level, while other identified long-term relationships are significant at the 1 % level. This underscores the less robustness and reliability of lnY ∼ lnET and lnET ∼ lnY relationships. The presence of a (conservative) bidirectional long-term relationship between economic output and energy consumption in the transportation sector reflects the intricate and multifaceted nature of the relationship between these two variables. Final energy consumption in the transportation sector predominantly involves gasoline and diesel consumption. Gasoline is primarily used for private cars and taxis, while diesel is mostly consumed for freight transportation in Iran. The implication here would be that while passenger transportation is influenced by income levels, the role of freight transportation in contributing to economic output is distinct and driven by different factors.

Furthermore, the study results reveal that when GDP is modeled as a function of total final energy consumption, capital, labor, and trade openness, the null hypothesis of no long-run relationship cannot be rejected. This underscores the significance of examining the explanatory power of sectoral energy consumption concerning economic output.

In alternative specifications where energy is replaced with exergy, the overall results remain largely consistent with the main analysis. However, there are two exceptions: the specifications lnEx ∼ lnY and lnY ∼ lnExT, fail to pass either the F-statistic test or diagnostic tests. Comparing the F-statistic and adjusted R-Squared for paired specifications, it becomes evident that exergy and energy exhibit similar explanatory power with respect to economic output. In essence, the results reject the hypothesis that exergy provides a superior explanation for economic output compared to energy. This implies that, in the context of this study, the choice between exergy and energy as explanatory variables does not significantly impact the understanding of their relationships between energy and economic output.

The findings in this study regarding the long-term relationship between total final energy consumption and economic output align closely with the conclusions reached in prior research by M. Zamani [19], M. Mehrara [22], N. Apergis and C.F. Tang [23], C.C. Lee and C.P. Chang [24], S. Nasreen and S. Anwar [25], and M.R. Lotfalipour et al. [27]. However, they do not fully concur with the results of studies conducted by S. Erdogan et al. [29], M.S. Gorus, and M. Aydin [28], P.K. Narayan, and S. Popp [21]. These disparities in findings emphasize the complexity and multifaceted nature of the relationship between energy consumption and economic growth, which can be influenced by a multitude of factors and conditions specific to each study's context.

Regarding the long-run relationship between economic output and industrial energy consumption, the elasticity with respect to energy use is positive and significant (0.48). The positive and significant elasticity value shows that industrial energy use is a key factor in economic growth. The calculated elasticity is compatible with the values offered for developing economies [102].Research on developed countries [103] suggests that policies targeting energy consumption in such nations may have limited adverse effects on long-term economic growth. However, our study shows a different pattern in Iran, a developing nation.

Considering the findings of this study, which underscore the significant positive elasticity of industrial energy use on economic growth, countries like Iran face a daunting challenge in limiting their energy consumption to reduce emissions. The pursuit of policies aimed at reducing energy use and greenhouse gas emissions must be approached with careful consideration. Specifically, such policies need to be crafted and implemented in a nuanced manner to mitigate any potential adverse impacts. This is particularly crucial given the intricate relationship between energy consumption, economic growth, and environmental sustainability highlighted in our research. Therefore, the formulation and implementation of policies to reduce energy consumption and emissions require a delicate balance, considering the economic development imperatives while also addressing environmental concerns.

### 4.2 Causality relationships

In this analysis, the joint significance of the differenced explanatory variables of energy or GDP indicates the presence of short-run causality either from energy to GDP or from GDP to energy, respectively. Meanwhile, the t-statistic on the coefficients of the lagged error-correction term provides insight into the significance of long-run causality. The results of examining short-run and long-run dynamics are presented in Table 5.

**Table 5:** Causality analysis results.

Model specification	lag orders	ECT		Short-run causality	
		coefficient	Significance	Specification	Significance
lnY ∼ lnE + lnK + lnTO + lnL	(3,4,4,0,0)	–	–	lnY ∼ lnE	0.01
lnE ∼ lnY + lnK + lnTO + lnL	(3,3,3,3,0)	−0.14	1.48e-06	lnE ∼ lnY	0.04
lnY ∼ lnEI + lnK + lnTO + lnL	(4,3,2,4,2)	−0.38	8.27e-08	lnY ∼ lnEI	0.19
lnEI ∼ lnY + lnK + lnTO + lnL	(0,2,1,4,0)	–	–	lnEI ∼ lnY	0.04
lnY ∼ lnEB + lnK + lnTO + lnL	(3,4,4,2,1)	–	–	lnY ∼ lnEB	0.25
lnEB ∼ lnY + lnK + lnTO + lnL	(4,4,3,4,4)	−0.43	5.72e-06	lnEB ∼ lnY	0.15
lnY ∼ lnET + lnK + lnTO + lnL	(4,3,1,4,3)	−0.09	4.79e-06	lnY ∼ lnET	0.22
lnET ∼ lnY + lnK + lnTO + lnL	(4,4,4,3,2)	−0.04	1.26e-05	lnET ∼ lnY	0.09

The findings confirm the economically influential role of energy consumption in the industry and transportation sectors in the long run. Energy consumption in the buildings sector, despite constituting a sizable portion of total energy consumption, does not exhibit either a propelling or restrictive effect on economic output in the long run. Similarly, total energy consumption does not play a dominant role in determining long-term economic output; rather, it appears to be influenced by income. Moreover, income would also affect transportation energy use in the long run.

However, in the short run, variations in total energy consumption significantly impact changes in economic output, and total energy consumption is notably responsive to changes in economic output. Consequently, the study reveals the existence of bidirectional short-run causality between total energy consumption and economic output, aligning with the feedback hypothesis, highlighting the interdependent nature of this relationship in the short run [104]. Furthermore, income would affect both transportation and industrial energy use in the short term.

The application of the ECM approach reaffirms the results of the bounds testing approach. As required, ECT coefficients in the proposed dynamics are both negative and significant at the 1 % level. The error correction term coefficient is called the adjustment coefficient or speed of adjustment [105]. It illustrates how much of the adjustment to equilibrium takes place in each period. A ECT of −1 shows that the adjustment is instantaneous, or 100 % of the adjustment takes place within a year. Regarding the ECT coefficient for the lnY ∼ lnEI dynamic, which is −0.38, it will take approximately less than three years to reach long-run equilibrium in case of any shock or disturbance. The estimated adjustment speed values, as shown in Table 6, are comparable with those computed by other studies.

**Table 6:** Estimates of adjustment speed from this study and some other studies.

Type of relationship	Speed of adjustment (%)	reference
energy ∼ economic output	12	Current study
economic output ∼ industrial energy use	38	Current study
energy use in the buildings ∼ economic output	20	Current study
economic output ∼ energy use in the transportation sector	29	Current study
energy use in the transportation sector ∼ economic output	19	Current study
renewable energy consumptioñ economic growth	14	N. Apergis and J.E. Payne [9]
economic growth ∼ renewable energy consumption	13	
economic growth ∼ energy production from oil	58	N. Ahmad and L. Du [26]
economic growth ∼ energy consumption	36	S. Nasreen and S. Anwar [25]
energy consumptioñ economic growth	14	
electricity consumptioñ economic output in Western Europe	31	P.K. Narayan and S. Popp [21]
electricity consumptioñ economic output in Asia	34	
economic output ∼ electricity consumption in Asia	3	
electricity consumptioñ economic output in Latin America	37	
electricity consumptioñ economic output in Middle East	37	
electricity consumptioñ economic output in Africa	49	

**Fig. 3:**
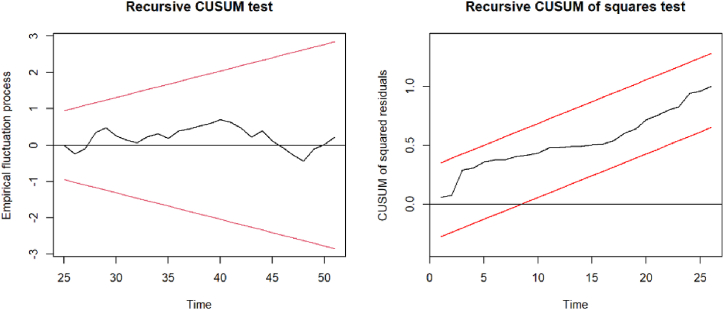
Plots of CUSUM and CUSUM-of-squares test results for lnY ∼ lnEI.

In addition to assessing normality, serial correlation, heteroscedasticity, and model specification, we rigorously examine the stability of coefficients in our analysis. This examination is achieved through the application of cumulative sums of standardized residuals and cumulative sums of squared standardized residuals, commonly referred to as CUSUM and CUSUM-of-squares tests [106]. These tests aim to detect any structural changes in the coefficients over time. If the paths of fluctuations cross the boundaries calculated for a specified significance level (usually set at 0.05), these fluctuations are considered statistically significant, which results in the rejection of the null hypothesis that no structural change has occurred. As demonstrated in Fig. 3 for the relationship between GDP and final energy consumption in the industry sector, similar checks for the entirety of the long-term relationships verify the stability of coefficients in the developed model specifications. This comprehensive approach ensures the reliability and robustness of the findings.

### 4.3 Implications from other case studies

Building upon the concept of productive energy use previously discussed, empirical analysis of the energy-economy relationship in Iran statistically validates this notion. The findings underscore that energy consumption in the industry and transportation sectors significantly propels economic output, particularly in Iran, characterized by its reliance on resource-based industries [107]. Moreover, the analysis highlights income as the pivotal factor shaping total energy consumption and buildings energy use. This distinction elucidates how varying income levels and sectoral energy usage dynamics intricately influence the energy-economy relationship, offering valuable insights within the Iranian context.

To bolster the robustness of our earlier findings and broaden the examination of selected sectoral energy consumption as production factors in globally utilized integrated energy and economy systems models, we conducted an analysis of long-term relationships between sectoral energy use and economic output across diverse countries. While prevailing integrated assessment models like MESSAGEix [108] typically employ a nested Constant Elasticity of Substitution (CES) production function with inputs of capital, labor, and all end-use energy (Equation (6)), our study suggests a potential refinement to this approach. Specifically, it proposes focusing on specific sectoral energy uses rather than encompassing all energy end-uses. Furthermore, our findings advocate for shifting the focus from summing all sectoral energy usage to aggregating productive energy end-uses. This refinement carries manifold implications: it enhances specificity in understanding the nexus between economic output and energy consumption, enables targeted interventions for enhancing energy productivity, streamlines Integrated Assessment Model (IAM) structures for heightened modeling precision, and facilitates tailored climate policies to address sectoral disparities [97].

6 Yt=(a*Ktρα*Ltρ(1−α)+∑sb*Etρ)1ρ

Here, Y_t_, K_t_, L_t_, and E_t_ represent GDP, capital stock, labor force, and sectoral energy use proxy variables, respectively. Additionally, ρ=ϵ−1ϵ where ϵ denotes the elasticity of substitution, α represents the capital value share parameter, a signifies the production function coefficient of capital and labor, and b denotes the production function coefficients of the various sectoral energy end-uses.

This cross-country analysis employs a methodology akin to that employed in the preceding phase of the research. While employing panel data analysis could yield more robust inferences, it is essential to acknowledge that due to data constraints, the analysis timeframe was confined to the period between 1971 and 2018 [97].

The results of the cross-country analysis, as presented in Table 7, compellingly support the proposed relationship between sectoral or total energy consumption and economic output. Particularly, the specification of lnY ∼ lnEI is found to hold in 12 out of 14 cases, while the specification of lnY ∼ lnE is only valid in 3 countries. Additionally, the specification of lnY ∼ lnET is observed to be valid in just 2 countries. Although both specifications of lnY ∼ lnE and lnY ∼ lnEI are applicable to Chile, France, and Korea, employing industrial energy consumption as the explanatory variable consistently leads to a higher adjusted R-Squared in these cases. These findings advocate for an alternative formulation for the production function within integrated assessment models, suggesting the adoption of industrial energy end-use instead of encompassing all energy end-uses. This reaffirms the pivotal role of industrial energy use in driving economic production. Based on the empirical results, the prolonged downturn in industrial value-added in the EU, despite the subsequent decrease in energy prices following the post-Russia war price hike, gains clarity, emphasizing the enduring impact of energy shortages in the industrial sector [109].

**Table 7:** The results of the cointegration analysis across different countries.

country	Specification^a^		Statistic value		
		F-statistic	BG statistic^b^	BP statistic^b^	Shapiro-Wilk statistic^b^
Austria	lnY ∼ lnE	5.422	0.487	0.955	0.033
	lnY ∼ lnEI	5.872	0.841	0.504	0.749
	lnY ∼ lnET	5.483	0.173	0.846	0.015
Chile	lnY ∼ lnE	4.744	0.424	0.950	0.108
	lnY ∼ lnEI	5.316	0.166	0.208	0.560
	lnY ∼ lnET	2.047	0.694	0.077	0.914
Colombia	lnY ∼ lnE	2.206	0.157	0.227	0.802
	lnY ∼ lnEI	1.543	0.753	0.528	0.163
	lnY ∼ lnET	2.838	0.084	0.711	0.973
Denmark	lnY ∼ lnE	4.335	0.316	0.965	0.853
	lnY ∼ lnEI	7.816	0.829	0.763	0.662
	lnY ∼ lnET	5.180	0.772	0.910	0.770
France	lnY ∼ lnE	11.267	0.673	0.531	0.970
	lnY ∼ lnEI	9.685	0.782	0.112	0.742
	lnY ∼ lnET	9.190	0.931	0.054	0.943
Germany	lnY ∼ lnE	3.780	0.642	0.209	0.468
	lnY ∼ lnEI	7.633	0.442	0.428	0.992
	lnY ∼ lnET	2.815	0.829	0.669	0.523
Iran	lnY ∼ lnE	1.476	0.158	0.023	0.015
	lnY ∼ lnEI	5.789	0.855	0.213	0.194
	lnY ∼ lnET	2.749	0.587	0.029	0.424
Italy	lnY ∼ lnE	12.199	0.082	0.918	0.647
	lnY ∼ lnEI	15.965	0.103	0.344	0.524
	lnY ∼ lnET	9.479	0.415	0.447	0.033
Japan	lnY ∼ lnE	4.239	0.684	0.424	0.832
	lnY ∼ lnEI	6.954	0.433	0.845	0.923
	lnY ∼ lnET	3.707	0.761	0.793	0.321
Korea	lnY ∼ lnE	8.773	0.234	0.753	0.760
	lnY ∼ lnEI	6.773	0.233	0.184	0.644
	lnY ∼ lnET	6.632	0.146	0.142	0.526
Mexico	lnY ∼ lnE	4.117	0.274	0.075	0.777
	lnY ∼ lnEI	5.619	0.070	0.010	0.964
	lnY ∼ lnET	9.598	0.139	0.303	0.101
Portugal	lnY ∼ lnE	2.031	0.338	0.029	0.624
	lnY ∼ lnEI	2.828	0.797	0.660	0.830
	lnY ∼ lnET	2.969	0.314	0.447	0.675
Spain	lnY ∼ lnE	3.206	0.649	0.360	0.881
	lnY ∼ lnEI	5.077	0.746	0.566	0.228
	lnY ∼ lnET	3.279	0.944	0.671	0.797
United States	lnY ∼ lnE	8.227	0.4161	0.0285	0.6815
	lnY ∼ lnEI	7.978	0.3753	0.3729	0.7117
	lnY ∼ lnET	7.581	0.501	0.05551	0.1879

a All the specifications consider capital, labor, and trade openness as independent variables besides the mentioned one.

b in p-values.

The cross-country findings concerning Iran underscore that energy consumption in the industry sector is the sole energy-related factor capable of explaining economic growth. It differs slightly from the earlier results where energy use in the transportation sector was also explaining; however, the F-statistics value of the lnY ∼ lnET model for Iran reported in Table 5 is not significantly higher than the upper bound critical value at 5 percent. The observed disparities in results, could be attributed to variations in the timeframes and data sources. The earlier examination of the long-term relationship between energy and the economy in Iran was based on data derived from national databases over an extended period. In contrast, the cross-country analysis relies on internationally recognized and available data, which may contribute to differences in the outcomes.

## 5 Conclusion and policy implications

The current investigation stands as a distinctive research endeavor delving into the intricate relationship between economic output and sectoral energy consumption, an exploration that extends beyond the ambit of total energy consumption or energy use by resource type. This study seeks to probe this complex relationship considering the influence of key control variables such as capital stock, labor force, and trade openness. Employing a methodological framework comprising a multi-step approach, the study unfolds as follows: (1) conducting stationarity tests for unit root, (2) undertaking bounds testing for cointegration, and (3) executing a comprehensive causality analysis, utilizing the ARDL ECM method. This systematic method emphasizes its relevance in furthering our understanding of the linkages between economic production and energy use in Iran, guiding the formulation of effective policy measures.

While a conclusive long-run relationship has been ascertained, extending from economic output to total energy consumption for Iran, the converse causal link is not substantiated by meaningful empirical evidence. This finding challenges the growth hypothesis positing that total energy consumption significantly contributes to economic growth. The rejection of this hypothesis implies that energy conservation-oriented policies may not hinder economic growth in general [104].

Furthermore, our empirical findings reveal a pronounced degree of heterogeneity in the intricate relationships between sectoral energy consumption and economic output. Particularly noteworthy is the emergence of energy consumption in the industrial sector as the most pivotal determinant in explaining aggregate economic output over the long term. This observation supports the growth hypothesis, indicating that industrial energy consumption plays a crucial role in economic growth, both directly and as a complement to labor and capital in the production process. Consequently, this underscores the significant repercussions of indiscriminate energy-related policies and measures concerning energy provisioning for industrial sectors. Such measures, if not well-calibrated, have the potential to exert adverse effects on overall economic performance. Thus, our study strongly advocates for a reevaluation of current energy management plans in Iran, especially in light of recent measures to limit energy supply to industries in favor of households. Based on empirical evidence demonstrating the significant role of industrial energy consumption in driving economic production, it is evident that such plans may pose substantial risks to the economy in the long and short term. Therefore, there is a pressing need to replace these measures with energy efficiency-focused policies and demand-side management strategies.

The study reveals a significant industrial energy use elasticity value of 0.48 in Iran, which differs from the values observed in developed countries, where the link is weaker. This significant elasticity value emphasizes the critical challenge for countries such as Iran in reducing energy consumption to mitigate emissions while sustaining economic growth. Iran's consistent pursuit of a resource-based industrialization approach in recent decades has led the country to be significantly reliant on energy-intensive industrial operations. Our study's implications resonate with a clear message: the continued and assured supply of energy resources for industries plays a pivotal role in sustaining GDP growth. However, this guarantee comes at the cost of environmental concerns, given Iran's predominant reliance on fossil fuels for almost 99 % of its energy supply [2]. In response, policymakers face a pressing imperative to align with alternative energy supply and demand systems, as the current carbon-intensive energy supply model appears inherently incompatible with long-term sustainable development. Thus, the transition to renewable energy sources integrated into the industrial energy mix, alongside enhancing overall energy efficiency, emerges as an imperative strategic move in pursuit of a sustainable energy-environment-economy equilibrium.

Moreover, the study's findings bolster a unidirectional inference of long-term causality, pointing from economic growth to final energy consumption of buildings. This unidirectional relationship implies that energy conservation policies, particularly those encompassing demand management measures within the building sector, are unlikely to adversely affect GDP. This observation also underscores the evidence that higher income levels are positively associated with enhanced well-being and energy use in buildings. In essence, our study's results advocate for fostering energy-efficient practices within the building sector to harmonize with the overarching goal of sustaining economic growth and enhancing the quality of life.

The heterogeneous outcomes witnessed across various sectors highlight the pivotal significance of productive energy utilization in fostering economic growth. In this regard, industrial energy consumption emerges as productive energy usage, given its direct impact on economic output. In stark contrast, energy consumption within the buildings sector is primarily geared towards providing comfort and well-being services that, while integral to overall quality of life, are not intrinsically tied to economic productivity.

The empirical findings derived from an examination of the dynamics within the context of Iran find resonance within the broader scope of panel data analysis. The elucidated long-term relationships between economic output and sectoral energy consumption, based on data encompassing fourteen diverse countries, reaffirm the centrality of industrial energy consumption as a key driver of economic production. In over 85 % of scrutinized economies, industrial energy consumption emerges as a pivotal factor in explaining variations in economic growth, whereas total energy consumption falls short of meeting the criteria for designation as a right production factor in almost 80 % of case studies. This observation bears significant relevance within the context of integrated assessment models employed in devising strategies for climate change mitigation. It signals the necessity for a reevaluation of the underpinning formulations within such models, with the potential to yield different robust results. The fundamental implication is that the role of industrial energy consumption as a productive energy use driver has far-reaching implications for understanding economic growth dynamics, calling for a reexamination of the models informing climate change mitigation strategies.

In an alternative analytical framework, the conventional measure of energy is substituted with exergy to scrutinize its explanatory power concerning economic growth. A comparative examination of the results shows that both exergy and traditional energy use exhibit similar qualifications in elucidating economic output variations, effectively countering the notion that exergy might serve as a superior explanatory variable concerning changes in economic production.

Furthermore, the findings and discussions, particularly regarding the dynamics between economic output and energy use in the buildings and transportation sectors, underscore the novel approach to energy and climate policies, advocating a shift towards focusing on energy services rather than mere energy consumption. The findings of the study reaffirm the need for innovative policies that center on redefining energy use within the context of service provision [110]. For instance, considering mobility not as energy consumption but as a service derived from energy utilization opens avenues for transformative policy interventions. Crafting effective policies to achieve this delicate balance requires meticulous attention to avoid adverse impacts. It underscores the necessity for precise policy formulation and implementation to address both economic and environmental imperatives effectively.

Replicating this analysis using diverse analytical methods and alternative productivity proxy variables across a more extensive spectrum of countries and a longer temporal horizon would undoubtedly yield more robust and generalizable implications, particularly as they pertain to the methodological advancement of integrated assessment models. In addition, future empirical inquiries might consider introducing variables related to economy structure or environmental externalities [111] to dissect the intricate interplay among energy, economy, and environmental factors, particularly regarding the cross-country analysis studies. It would be an attempt to bridge the energy consumption and economic output literature with the literature on the relationship between economic output and the environment.

## Data availability statement

The authors assert that all the essential data supporting the study's findings are already encompassed within the article itself, with no additional required data beyond what is presented.

## CRediT authorship contribution statement

**Hesam Ghadaksaz:** Writing – review & editing, Writing – original draft, Visualization, Validation, Methodology, Investigation, Formal analysis, Data curation, Conceptualization. **Yadollah Saboohi:** Writing – review & editing, Supervision, Methodology, Conceptualization.

## Declaration of competing interest

The authors declare that they have no known competing financial interests or personal relationships that could have appeared to influence the work reported in this paper.
